# Free surgery for CHD through philanthropy—a sustainable model?

**DOI:** 10.1007/s12055-024-01813-7

**Published:** 2024-10-22

**Authors:** Prabhatha Rashmi Murthy, Sowmya Jandhyala, Shaun Prasanth Setty, Sreenivas Chodagam

**Affiliations:** 1 Sri Sathya Sai Sanjeevani Center for Child Heart Care, Navi Mumbai, Maharashtra India; 2 Sri Sathya Sai Sanjeevani Centres for Child Heart Care, Bengaluru, Karnataka India; 3 Miller Children’s Hospital, Long Beach, CA USA

**Keywords:** Congenital heart disease, CHD, Free healthcare, Philanthropy, Sustainability, GIVE model, India, Sri Sathya Sai Sanjeevani, Low-middle income countries

## Abstract

Among the multiple challenges faced by children from low- and middle-income countries (LMICs) with congenital heart disease (CHD), the economics of care remains foremost, contributing significantly to morbidity and mortality. This paper evaluates the four existing finance models available for healthcare systems and proposes a new model—the GIVE model (government, institutions and individuals, values, and engagements)—as the fifth model for global sustainable healthcare systems. The paper presents an evaluation of a chain of three paediatric cardiac hospitals in India to assess the sustainability of their philanthropy-based operational model, through which surgeries are offered completely free of cost to children with CHD. The three Sri Sathya Sai Sanjeevani Centres for Child Heart Care in India have been proponents of this philanthropic model for over a decade. From February 2013 to January 2024, 19,684 patients with CHD received surgeries at no cost. The average cost of surgery was reported to be USD (United States Dollar) 1800. A case study of one of the Sanjeevani Centres showed that 23.8% of patients were in STAT Category 3 and above, as defined by the Society of Thoracic Surgeons-European Association for Cardio-Thoracic Surgery, with an in-hospital mortality rate of 2.08%. The evaluation highlighted the institution’s numerous strategies to enable sustainability in key aspects of operations, economics, and social impact. Despite challenges, the Sai Sanjeevani philanthropic model, which encompasses both economic and social impact, is dependable and can be replicated. The proposed GIVE model is recommended for adoption by LMICs as a global way forward to enable free CHD surgeries through sustained philanthropy, strengthened by a shared vision and collaborations.

## Introduction

Universal health coverage (UHC) aims to ensure equity in access to healthcare, provide a comprehensive range of need-based services, and shield individuals from the economic burden associated with seeking medical care. The explicit inclusion of UHC in the United Nations (UN) Sustainable Development Goals (SDG) Goal 3–Good Health and Well-being and World Health Organization (WHO) programmatic objectives emphasises its heightened focus in global policies [1]. World Health Organization and World Bank published measurement framework identifiers—promotion, prevention, treatment, rehabilitation, and palliation as the services to be included to ensure UHC [2]. With over 150 million (M) individuals annually estimated to be pushed to poverty around the world, access to healthcare and UHC is an issue which is far from addressed, particularly in lower to middle Sustainable Development Index (SDI) countries [3]. In the Indian context, 62% dependence on the private sector for healthcare services exacerbates accessibility issues, especially for low-income individuals. Rural India is grappling with inadequate infrastructure and shortage of medical and nursing professionals and thus continues to experience poor access to healthcare services [4, 5].

## Congenital heart diseases—unveiling global challenges

Congenital heart diseases (CHD) account for one-third of all congenital birth defects [2, 6]. The global incidence of CHD varies, but it is estimated that approximately 8 to 12 in 1000 live births globally or 1.35–1.4 M infants are born every year with CHD, of which 25% die in their first year [6–10]. The prevalence number is more alarming at close to 12 M [6].

A status report on availability, access, and funding across 193 countries highlights insufficient or intermittent care in more than 75% of the countries, particularly across most parts of Africa, Asia, and South America. Lower SDI countries are found to be reporting higher CHD mortalities, indicating a direct relationship between income levels and CHD mortalities [7, 11]. A marked variation is also reported on the costs of paediatric cardiac surgeries estimated to be between United States dollar (USD) 800–6000 in India, USD 10,000 in Africa, and between USD 12,000 and 50,000 in the USA (United States of America), with out-of-pocket expenditure as the most prevalent financing option [8, 12].

## Tiny hearts, big challenges—the India story

The India Newborn Action Plan emphasises focus on child survival programs, systems, and delivery platforms as an essential step towards the country’s commitment to child health [13]. It is estimated that each year in India, around 240,000 children (8 out of 1000 live births) are born with CHDs [13, 14]. Congenital heart disease–related mortality accounts for close to one-third of all mortalities due to congenital defects and less than 5% of babies born with critical CHD undergo receive definitive treatment [15, 16]. This indirectly contributes to deaths due to birth defects, which are the third leading cause of neonatal mortality in over 70% of Indian states [17].

### Demand–supply gap

Of the total CHD children born each year, about one-fifth require early intervention in the first year to survive. The burden staring at the country is grave with the annual paediatric cardiac surgeries conducted being much lesser than the estimated prevalence of 2 M children with CHD [6, 18]. Close to 150 practicing paediatric cardiac surgeons address this large problem, which falls short of the estimate that the country needs 300–500 paediatric cardiac programs or 1000 paediatric cardiac surgeons to meet accessibility requirements [12, 19].

### Access inequities

Geographical disparities further exacerbate the challenges impacted by factors including varying birth rates, concentration of paediatric cardiac services in South India, delayed diagnoses, and limited access to timely interventions in rural India [14].

### Unaffordable care

Most centres caring for CHDs are in the private sector and are therefore not affordable for most of the Indian population. Very few studies attempt to estimate the number of families who painfully wait for some financing option as their little one continues to grow with increasing morbidities. With India’s per capita net national income (NNI) being less than USD 1200, paediatric cardiac surgeries are unaffordable by most families [20]. Among those who access care, a shocking 35% of paediatric cardiac surgeries are funded by families themselves, 40% by government schemes, 20% by hospitals run by charitable and non-government organisations, and 5% by other ad hoc financing programs [14]. This need supply gap is also reflected in the huge waiting lists under charitably run programs.

## The need for free CHD surgeries: an investment not an expenditure

Access to free paediatric cardiac surgeries is both a moral duty and an economic necessity. It reflects a society’s commitment to its vulnerable members and prevents long-term consequences of untreated CHDs, fostering a generation vital for economic growth. The trauma of not being able to save one’s child is a poor statement of a society or a nation’s humaneness. A CHD child is rightly described ‘a child whose name is Today’, for many a time it has no tomorrow.

The inability to afford surgical expenses can strain finances, impacting essential needs like education and housing. Conversely, providing free surgeries reduces economic burdens on families and society, with each surviving child potentially contributing significantly to national income, making free cardiac care a societal imperative. A study conducted in low-middle income countries (LMICs) for cost-effectiveness of humanitarian missions for free CHD surgeries reports that each surviving child potentially gains 39.9 disability-adjusted life-years averted and potentially contributes USD 159,533 in gross national income per capita at purchasing power during his or her extended lifetime [21].

## Exploring financing models for affordable paediatric cardiac care

To evaluate the need and relevance of sustainable philanthropy for CHD care, it is essential to examine current models of healthcare systems. Four prominent models have evolved over decades to meet varying patient and system needs [22].

### Beveridge model

Where healthcare services are publicly funded and provided, it is relevant to CHDs, but often in low SDI countries it faces challenges of mid to severely inadequate infrastructure, insufficient skilled specialists, insufficient medical supplies, and compromised quality.

### Bismarck model

Where healthcare services rely on mostly mandatory social insurance contributions/premium, it is found challenging in the context of middle and low SDI countries, including India, which do not implement the model due to employment and affordability issues.

### National health insurance

Where elements of both Beveridge and Bismarck models are combined, it has the potential of offering comprehensive coverage for CHD patients, by balancing public and private strengths to meet the goals of national paediatric cardiac care. Two examples stand out in the context of Indian Child Health.

- *Rashtriya Bal Swasthya Karyakram (RBSK)*—a flagship scheme of the Government of India, launched in 2013, which focusses on identification, prevention, and management and medical, surgical, and therapy-based, child health and also empanels healthcare institutions to provide care for screened patients across 32 conditions, including congenital defects [14, 23, 24].
- *Ayushman Bharat and state-run programs*—launched in 2018, the Pradhan Mantri Jan Arogya Yojana (PM-JAY), a part of the Government of India’s Ayushman Bharat Program, is the world’s largest health insurance scheme, providing financial protection to close to 500 M beneficiaries, including 107.5 M poor, deprived rural families and other identified underprivileged workers [25, 26]. With regard to CHDs, the program covers a wide range of CHD presentations through empaneled hospitals, covering all the costs. However, low package bands (ranging between USD 350 and 1500 based on the condition), reimbursement delays, and state-level implementation variations continue to be challenges [27, 28]. Certain states also run dedicated CHD programs towards increased reach.

### Out-of-pocket

Which is estimated at an alarming 62.6% of overall healthcare expenditure in India, impacting households and underscoring the need for comprehensive insurance coverage [29].

## Introducing the GIVE model—the 5th model for a sustainable healthcare system

Healthcare systems globally have made significant clinical progress and increased availability of care but struggle to adequately meet the burden of paediatric cardiac issues, evident in the prevalence of CHD. Addressing this challenge necessitates a paradigm shift. This paper defines a new approach—the GIVE model that integrates key strengths of the current models and builds on them (Table 1). At the core of the GIVE model lies the acknowledgment that ‘giving is intrinsic to human nature’. It stems from the virtue of compassion, going beyond the nuances of a balance sheet.

**Table 1:** New proposed model for sustainable healthcare

G	Governments	Prioritise CHD care in national child health policy Define national frameworks for CHD screening, treatment, research, training, and philanthropy Strengthen public infrastructure Implement engagement programs with private and charitable organisations to address more children
I	Institutions and individuals	Build institutions of healthcare excellence for comprehensive CHD care Prioritise CHD philanthropy—infrastructure, screening, treatment, research, training, advocacy, and awareness programs Offer infrastructural, medical, strategic, and operational voluntary services towards strengthening CHD programs
V	Values	CHD institutions, programs, and healthcare professionals exemplifying social values of love, compassion, equality, and service Shift from human capital to human development approach [30]
E	Engagements	Strategic instruments for universal CHD care Collaborative multistakeholder engagements between governments, institutions, and the society Value creation goals, impact outcomes, and pathways Informed policy, implementation, advocacy, learning, research, and funding [31]

## Philanthropy in CHD care

The evolution of philanthropy in CHD has shifted from sporadic donations to comprehensive support, focusing on underserved populations. Specialised teams from developed countries conduct surgeries and aid capacity-building in LMICs through humanitarian missions, benefiting children, training local teams, and strengthening healthcare systems [32]. Some organisations prioritise awareness campaigns, long-term support programs, and innovation in diagnostics and treatment through translational research. This facilitates the creation of a shared vision between socially motivated individual and institutions creating a collaborative and sustainable model which fits into the GIVE framework.

## What is sustainability?

Every model, more in the healthcare space, needs to be scalable and sustainable. Sustainability is often viewed by policy makers, economic experts, and societal influencers through the prism of finance alone. Numerous studies on sustainability have defined social sustainability as the process of addressing social concerns while confronting risk, and an integration of social values and economic risk [33, 34]. However, there is a need for more systemic reviews on social sustainability [35].

Over the years, it is established that the social sector is a non-negotiable partner in achieving global sustainable development goals [35]. With regard to CHD care in India, charitable or non-governmental organisations already contribute to 20% of all care provided. This paper aims to evaluate the proposed GIVE model and its sustainability, defined as social impact and economics, through the study of Sri Sathya Sai Sanjeevani Centres for Child Heart Care, India.

## The Sri Sathya Sai Sanjeevani story—exemplifying sustainable philanthropy

### Twenty-nine thousand twelve free CHD surgeries and interventions in 11 years—face of the unknown Indian child

#### Background

**Fig. 1:**
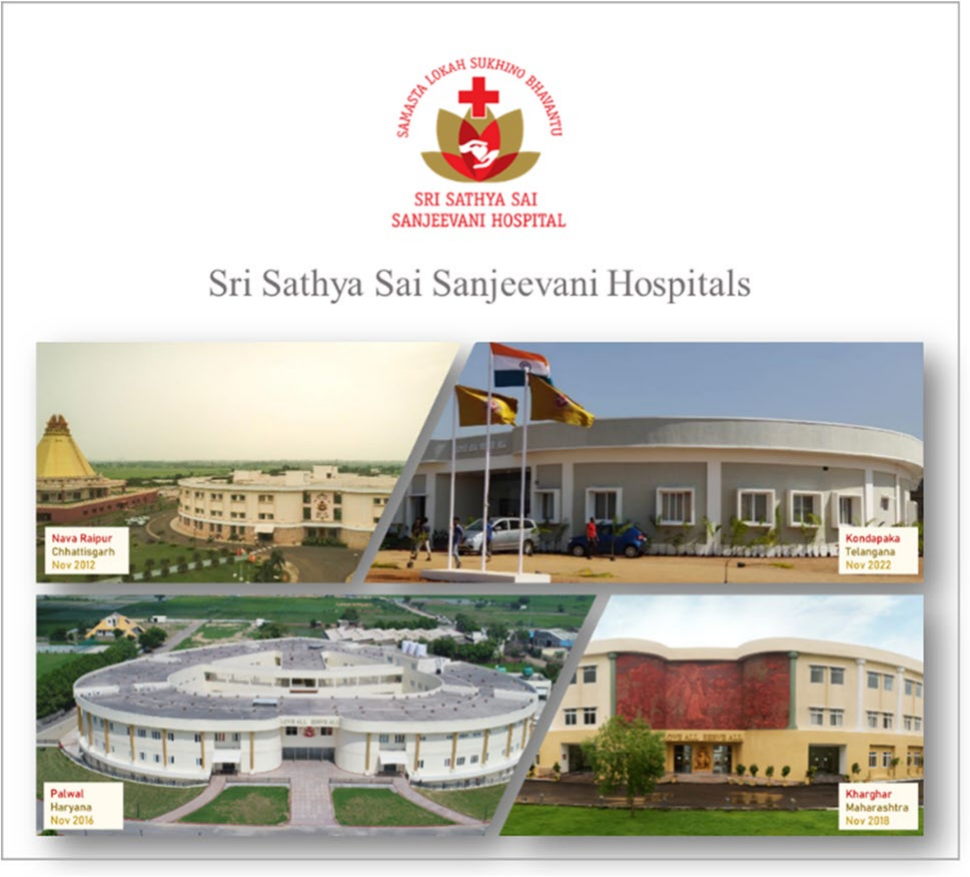
Sri Sathya Sai Sanjeevani Centres for Child Heart Care

Sri Sathya Sai Sanjeevani Hospitals (SSSSH) in India commenced their journey in 2012 with a vision to serve the cause of ‘Right to Healthy Childhood’ through creation of a holistic ecosystem resulting in reducing the global CHD burden. The first Centre for Child Heart Care was set up in Nava Raipur, Chhattisgarh, India, in November 2012. In November 2016, the second Sri Sathya Sai Sanjeevani International Centre for Child Heart Care & Research was inaugurated in Palwal, Haryana, and the third Centre was set up in Navi Mumbai, Maharashtra, in 2018. The fourth centre is set to commence paediatric cardiac surgeries in the state of Telangana in 2024 (Fig. 1).

Today Sai Sanjeevani Hospitals are the largest dedicated centres providing totally free of cost paediatric cardiac care for children with CHDs. The hospitals provide the entire spectrum of paediatric cardiac services to families accessing care including outpatient consultations, echocardiography (ECHO), radiology, pre-admission care, surgery/catheter-interventional procedures, post operative care, laboratory investigations, medicines, nutritional supplements, and counselling services. All these services are provided totally free of cost to all. To alleviate the financial strain on families with limited income, the hospital also provides complimentary accommodation and meals to patient attendants for the entire duration of treatment on the hospital premises.

#### Infrastructure and impact

‘Healthcare for the poor does not mean poor healthcare’

The SSSSH are equipped with state-of-the-art technology ensuring the children receive the best medical attention. The centres are staffed by an exemplary team of paediatric cardiac specialists, medical professionals, skilled nursing staff, and paramedical teams, deeply committed to serving the needs of the children seeking care (Table 2).

**Table 2:** Infrastructure of Sai Sanjeevani Centres for Child Heart Care

Paediatric cardiac operation theatres (OT)	10
Paediatric cardiac cath labs	3
Dedicated beds	300
Paediatric cardiac intensive care unit (ICU) beds	145
Specialists	38
Manpower: medical, nursing, paramedical, administrative, and support staff	902

As of 31 January 2024

For the period Feb 2013 to January 2024, the three operational centres managed 245,343 paediatric cardiac outpatients and conducted 29,012 procedures—19,684 paediatric cardiac surgeries and 9328 paediatric cardiac catheter-interventions from all states of India and 12 developing countries. Table 3 categorises the surgeries conducted into the STAT risk categories defined by the Society of Thoracic Surgeons-European Association for Cardio-Thoracic Surgery; a tool used to analyze risk of mortality for paediatric cardiac surgeries [36].

**Table 3:** STAT classification of CHD surgeries conducted by Sai Sanjeevani Centres, Feb 2013–June 2023 [36]

Overall mortality	1.38%
STAT 1	47.1%
STAT 2	44.8%
STAT 3	1.9%
STAT 4	6.2%
STAT 5	0.0%

#### Comprehensive approach to CHD

Sri Sathya Sai Sanjeevani acknowledged the necessity of embracing a comprehensive strategy for managing CHDs, by implementing a multifaceted approach, incorporating diverse initiatives focused on prevention, early detection, treatment, training, and research to pilot an ecosystem approach to address the prevalence of CHD.

##### Mother and child health (MCH)

Recognising the interconnected health of both MCH, the first totally free of cost Mother & Child hospital was set up in Nava Raipur (Chhattisgarh) in 2021. Upon the invitation of governmental, social, and private parties across states, four more Mother & Child hospitals were operationalised in a span of 1 year in Palwal (Haryana), Yavatmal (Maharashtra), Raiwala (Uttarakhand), and Jamshedpur (Jharkhand). The hospitals provide the entire spectrum of maternal care for rural and tribal pregnant women. Free fetal ECHO services are also offered by the Departments of Pediatric Cardiology at the centres, particularly for the detection of complex anomalies and to enable informed pregnancy decision based of probable postnatal outcomes [37, 38]. The hospitals also work closely with district administrations to integrate national- and state-level maternal health programs like the Janani Suraksha Yojana, Janani Shishu Suraksha Yojana, and ASHA (Accredited Social Health Activist) program.

##### Screening

To address the estimated prevalence of CHDs in schools (3.8 out of 1000 school children [39]), the hospitals initiated the Divine Mother & Child Health Program in September 2017 which is a comprehensive antenatal and CHD screening program for rural children. This is a comprehensive early detection and systemic health screening initiative based on the Government of India’s RBSK program. Dedicated outreach programs screen children for CHDs in government-run anganwadi centres (rural child care centres) and schools and refer them to Sai Sanjeevani Hospitals for confirmatory ECHO tests and treatment, as required [40]. The number of children screened for CHDs from September 2017 to January is 206,527.

##### Training

The availability of paediatric cardiac specialists, nursing, and paramedical teams is considered a major barrier in ensuring paediatric cardiac care. The hospitals conduct numerous academic, training, and skill development programs for doctors, nurses, and allied healthcare providers to contribute to national capacities and inspire young specialists to expand paediatric cardiac services in the country. All courses offered by the hospitals are again totally free of cost, thus removing any financial barriers for bright medical and paramedical providers to get trained. Some of the courses offered include:

- Doctorate of National Board (DrNB) in Pediatric Cardiology under the National Board of Examinations—India, Institutional Fellowships in Paediatric Cardiac Surgery, Paediatric Cardiac Anaesthesia, and Paediatric Cardiology—46 specialists trained.
- Masters in cardiothoracic nursing by the Sri Sathya Sai Sanjeevani Institute of Nursing & Allied Healthcare Sciences—53 nurses trained.
- Specialised training for nursing, paramedical, and technical staff (including critical care nursing, ECHO technicians, X-ray technicians, lab technicians, OT assistants, physician assistants, physiotherapists, perfusionists, and bio-medical engineers)—187 nurses and technicians trained.

##### Research

The Sri Sathya Sai Sanjeevani Research Centre for CHDs was set up in 2018 to understand and contribute to ‘life’ through the pursuit of advanced human genomic research in CHD. Housing two biobanks with over 6000 heart tissue and blood samples, the Research Centre, run by dedicated researchers, envisions to do collaborative work on finding causes and solutions to address the issue of CHDs.

#### The case of Sai Sanjeevani, Kharghar, Navi Mumbai, Maharashtra, India

To get a more comprehensive view on patient care parameters, the full journey of Sri Sathya Sai Sanjeevani Centre for Child Heart Care, Kharghar, Navi Mumbai, Maharashtra, is being discussed. The centre commenced outpatient services in November 2018. With basic infrastructure including one operating room and a six-bedded ICU, the first surgical procedure was performed in May 2019. By the end of the year, 125 free CHD surgeries were conducted with no mortality. As work progressed amidst scaling of infrastructure and manpower building, clinical care was affected by the pandemic with Mumbai being one of the worst affected cities of India during the coronavirus disease (COVID-19) pandemic. This was in line with the trend seen across 24 paediatric cardiac centres in India which reported 66.8% reduction in inpatient admission between 2019 and 2020 [41]. Soon services reopened with strict protocols and patient testing, amidst restricted travels for patient families and supply chains for consumables. Soon the centre expanded to a new OT—Cath lab—ICU complex, with two operating rooms, one cath lab, and a 26-bedded cardiac ICU.

**Fig. 2:**
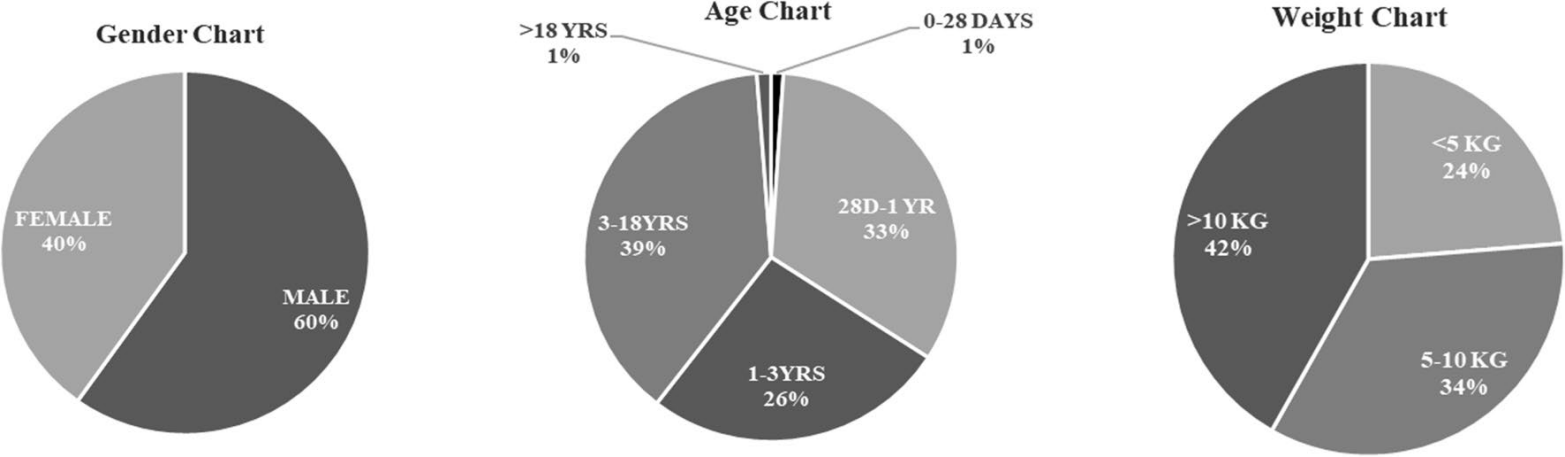
Gender, age, and weight distribution of 2541 CHD surgeries in Sai Sanjeevani, Kharghar (May 19–Jan 24)

Analysis of retrospective data of the hospital highlights that 2541 paediatric cardiac surgeries were conducted by the hospital in the period May 2019–January 2024, all totally free of cost. This included both primary and redo procedures on the same patient (surgery for complication or revision of procedure or residual defects) or new staged procedures, but excludes reoperations for bleeding, pacemaker insertion, and wound debridement. Key areas in which patient data was captured were weight, age, gender, Society of Thoracic Surgeons (STS) category, cyanotics/acyanotics, morbidity, mortality, and ICU stay (Table 4; Table 5). The overall mortality rate was 2.08%. The average ICU stay across all STAT categories, inclusive of main and step-down ICU stay, was 2.65 days for 82% children, 7.29 days for 11% children, and 13.5 days for 7% children. Among the operated, 34.16% of children were neonates and infants and 23.73% were less than 5 kg (Fig. 2). The Kharghar Centre is currently revising its protocols to share data with the International Quality Improvement Collaborative (IQIC). Going forward, the Sai Sanjeevani centres will have a joint database, with other like-minded programs, capturing the data of the all children who have received treatment thus far.

**Table 4:** Type of CHD surgeries conducted at Sri Sathya Sai Sanjeevani Centre for Child Heart Care, Kharghar, Navi Mumbai

Surgical procedure	Total cases performed	Mortality	% Mortality
ASD closure	341	0	0%
VSD closure	968	09	0.92%
TOF repair (annulus sparing)	219	0	0%
AVSD repair	47	02	4.25%
SAM excision	04	0	0%
PAPVC repair	58	0	0%
DCRV repair	04	0	0%
Pulmonary valvotomy	01	0	0%
CoA repair	24	0	0%
RSOVA repair	01	0	0%
TOF repair with TAP	412	11	2.66%
ALCAPA repair	04	0	0%
PDA ligation	51	01	1.96%
TV repair	06	0	0%
BDG	64	02	3.12%
AP window closure	14	0	0%
MV repair	03	0	0%
RVOTO relief	06	0	0%
ROSS procedure	03	0	0%
Atrial septation	06	0	0%
Aortic valve repair	02	0	0%
Cor-triatriatum	01	0	0%
Implantation of RPA-MPA	06	01	16.6%
Rastelli/Nikaidoh procedure	09	01	11.11%
ASO	12	02	10.8%
Fontan completion	06	01	16.6%
Double aortic arch repair	04	0	0%
Potts shunt	01	0	0%
RV-PA conduit	09	01	11.11%
TAPVC repair	115	06	5.21%
Truncus repair	11	03	27.27%
PA banding	05	0	0%
Aortic arch repair	14	01	7.14%
IVTR for DORV	10	01	10%
Senning repair	06	01	16.6%
Cone repair for Ebstein’s anomaly	08	0	0%
ASO + VSD closure	34	05	14.7%
Left atrial (LA) myxoma	02	0	0%
ASO + arch repair	01	0	0%
Double switch operation	02	02	100%
Norwood procedure	02	02	100%

List of surgeries placed in order of complexity, excludes redo procedures on the same patient (surgery for complication or revision of procedure or residual defects), *ASO* arterial switch operation, *CoA* coarctation of aorta, *RVOTO* right ventricular outflow tract obstruction, *RSOVA* ruptured sinus of valsalva aneurysm, *RPA* right pulmonary artery, *TAP* trans annular patch, *MPA* main pulmonary artery, *ALCAPA* anomalous left coronary artery from pulmonary artery, *ASD* atrial septal defect, *PDA* patent ductus arteriosus, *VSD* ventricular septal defect, *TV* tricuspid valve, *TOF* tetralogy of Fallot, *BDG* bidirectional Glenn, *AVSD* atrio ventricular septal defect, *AP* aorto pulmonary, *SAM* sub aortic membrane, *MV* mitral valve, *PAPVC* partial anomalous pulmonary venous connection, *TAPVC* total anomalous pulmonary venous connection, *DCRV* double-chambered right ventricle, *IVTR* intraventricular tunnel repair, *AV repair* aortic valve repair, *RV-PA conduit* right ventricle to pulmonary artery conduit

**Table 5:** Morbidities reported

Neurological deficits	1	0.04%
Renal failure	1	0.04%
Chylothorax	12	0.47%
Paralysis of diaphragm	4	0.16%
Blood stream infections	83	3.27%
Urinary infections	0	0.00%
Ventilator associated infections	6	0.24%
Deep sternal infections	6	0.24%
Re-intubations	21	0.83%

#### Assessment using the GIVE model

This section attempts to evaluate the SSSSH’s work using the GIVE model introduced in this paper as the fifth model for healthcare systems.

##### Governments

Ensuring that all citizens have access to essential health services without facing financial hardship is the principal responsibility of the government through policy and regulation, health financing, infrastructure and capacity development, public awareness, quality standards, and partnerships. Recognising that the vision of the SSSSH is aimed at achieving and strengthening the government’s efforts to achieve UHC for MCH, the hospitals work closely with the Government of India and respective state governments to integrate with existing MCH programs. Particularly for CHDs, the Rashtriya Bal Swasthya Karyakram of the Government of India aims to screen, diagnose, and treat CHDs also powered by the Ayushman Bharat. SSSSH have signed memorandums of understanding (MOUs) under RBSK with the states of Chhattisgarh, Odisha, Uttarakhand, Uttar Pradesh, Maharashtra, Telangana, and Haryana, thus becoming a referral centre for CHDs.

##### Institutions and individuals

Hospitals of this scale and vision is only possible through the vision and strategic direction of the leadership, which is provided by the three trustees and six members on the board of advisors, who are dedicated and inspired leaders with a commitment to provide solace to parents whose children suffer unfortunate condition, understanding that it is not a matter of choice, but it is the only choice [40]. It is a constant effort by the leadership to recruit like-minded medical, nursing, paramedical, and administrative staff members, passionate about giving back to the society. The model exemplifies individual-, team-, and organisation-level social entrepreneurship to address one objective of quality, sustainable and free of cost CHD care for the underprivileged.

The inception of Sai Sanjeevani Hospitals was marked by the generous philanthropic contributions from both individual and institutional donors within the community who supported the cause of accessible child healthcare. Sai Sanjeevani continues to run and grow due to the continued philanthropy—CSR and individual, having the shared vision of addressing national MCH. Those individuals, genuinely inspired by the success of the free healthcare model, made substantial contributions, thereby significantly contributing to the well-being and future health of numerous beneficiaries [40]. Additionally, long-term engagements and associations with vendors, volunteers, and medical fraternity contribute to quality of care, beneficiary experience, and cost efficiencies.

##### Values

The values of love and service drive the efforts of Sai Sanjeevani. The investment made by Sanjeevani in saving a child’s life goes beyond the money spent. A child’s life is more important and precious than the money involved in saving it. By saving the child, the hospitals believe they restore humanity and faith in each other, like we did in saving lives in COVID times. No questions were asked; only the will and means to save life made sense. The SSSSH give face and voice to the unknown Indian child by providing each child and their family the primary right of dignity to life.

Every centre has a dedicated impact assessment and counselling team for managing in-hospital queries and stress and long-term follow-up of children treated at Sanjeevani Hospital for CHD to track their progress, social impact, and transformation in a program called *Ab Zindagi Sanjeevani ke Saath* (*Life with Sanjeevani*). Every discharge at Sanjeevani is a special event. Every discharged family is explained about the support provided by society for their child, also highlighting their increased responsibility in taking special care of the child and ensuring that the child grows to live the values of giving back to society. This connection to each family reflected in the presence of over 3000 children coming together in Sai Sanjeevani, Nava Raipur, to light oil lamps in celebration of the centre’s 10th-year anniversary, setting a new record under the Guiness World Records.

##### Engagements

Powerful engagements are binding factors that can potentially enable innovative approaches, mobilise expertise, broaden resource access, and foster accountability. SSSSH have fostered numerous engagements across verticals to power collaborative intent, vision, and efforts to achieve their social goals. Some key engagements with the private and social sectors include:

- Training—National Board of Examinations, Maharashtra University of Health Sciences, Indian Nursing Council, Children’s HeartLink, SRM University, DY Patil University
- Research—National University of Singapore; Advanced Centre for Treatment, Research and Education in Cancer; SRM University
- Public Health—National Institute of Public Health and Research for Public Health

#### Evaluating sustainability of SSSSH

The sustainability of Sri Sathya Sai Sanjeevani model is being evaluated using two key parameters—economics and social impact.

##### Economics

**Fig. 3:**
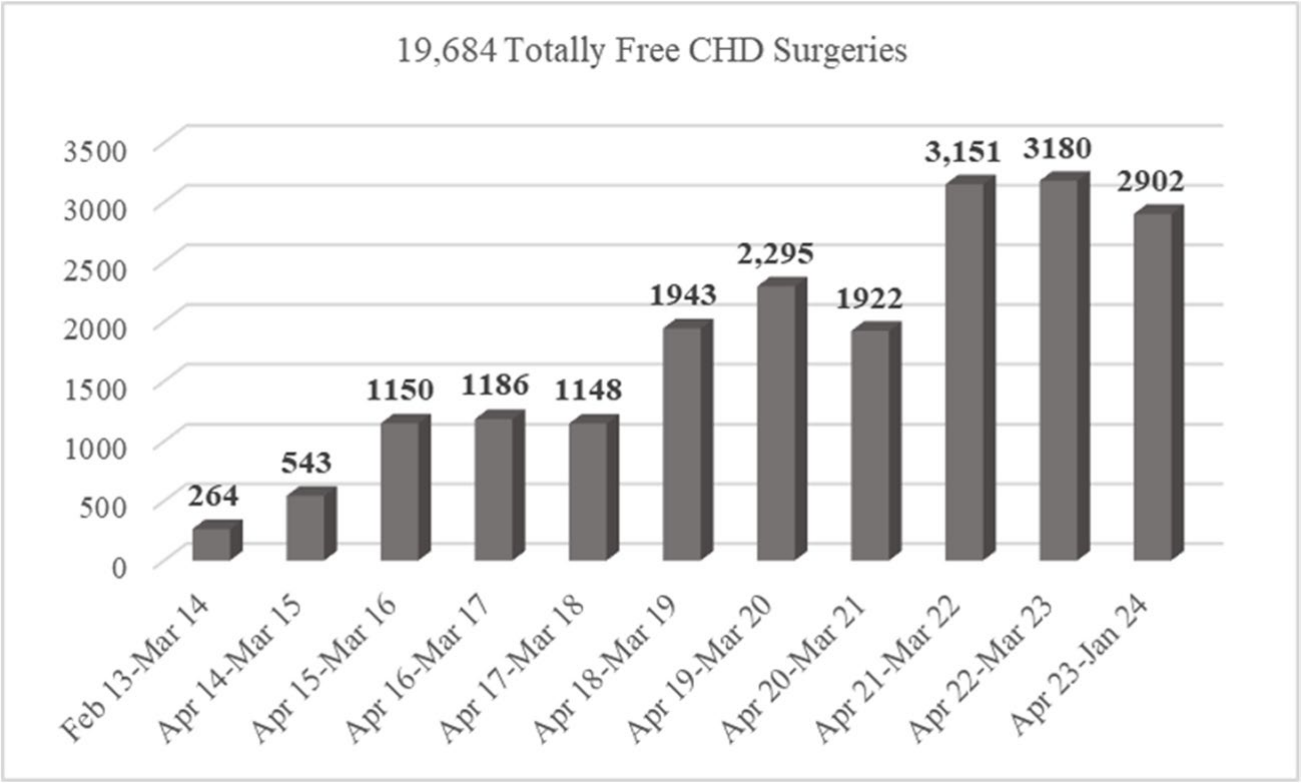
Graph of year on year growth of Pediatric Cardiac Surgeries at Sri Sathya Sai Sanjeevani Centres for Child Heart Care. Data considered up to 31 January, 2024

In times when profitability drives business models, SSSSH, in 11 years, have conducted over 29,000 paediatric cardiac surgeries and cath interventions, totally free of cost. The model has seen an increase in the number of CHD procedures conducted year on year reflecting its sustainability (Fig. 3).

#### Cost of services rendered

Since inception in 2012, SSSSH have raised and invested over Indian rupee (INR) 4.85 billion (B) (~ USD 58.4 M) in building hospital infrastructure (four child heart centres—three operational and one upcoming) and conducting paediatric cardiac surgeries, amounting to INR 2.95 B (~ USD 35.4 M), strongly establishing that the GIVE model is not just sustainable, but also scalable (Table 6).

**Table 6:** Calculating the cost of services rendered by SSSSH for the period Feb 2013–Jan 2024

Average cost of paediatric cardiac surgery at SSSSH*	INR 150,000 (USD 1800)
Total number of paediatric cardiac surgeries conducted for the period Feb 2013–Jan 2024	19,684
Total cost of child heart services	INR 2.95 B USD 35.4 M

*Inclusive of all services—outpatients, radiology, pathology, blood services, surgery, intensive care, inpatient care, physiotherapy, dietetics, food, and accommodation of families. Foreign exchange rates as of May 2024

The actual cost of surgery was reported to be INR 150,000 (USD 1800), based on the risk, comorbidities, age, weight, complications, and presence of major non-cardiac anomalies [40, 42]. SSSSH have managed to bring the costs down significantly by building economies of scale, operational efficiency, and sensitising hospital teams to avoid any wastage of resources, without any compromise on patient outcomes. With an expected increase in the number of cases in the coming years, the centres plan to monitor costs stratified by CHD category, age, and weight.

#### Philanthropy and corporate social responsibility (CSR)

Philanthropy in its traditional sense, donation of money by individuals and institutions for good causes, continues to be the binding factor between society and institutions.

In the Indian context, the country embarked on a bold experiment in mandating CSR for large companies, requiring spending at least 2% of profits in CSR, among other frameworks, through tightly defined and scrutinised processes by the Government of India [43]. As per published data by the Government of India—CSR portal, for the financial year 2021–2022, 19,043 companies participated in the CSR program with over 43,338 projects. The total national spend on CSR across 14 development areas for the aforesaid period was INR 262.78 B (~ USD 3166 M) [44]. The soul of this act is the responsibility for corporates to give back to society what they have gained from it to address poverty, education, health, sanitation, skill, water needs, among others [45]. Some key aspects of the CSR program that are encouraging for institutions attempting to provide free CHD care and healthcare are [46]:

- % of companies have healthcare as first priority—26%
- % companies prefer institutions with government partnerships—55%
- % companies prefer 3-year project durations—64%
- % of companies prefer sustainable implementing agencies—55%

With regard to SSSSH, a study of the trust’s annual financial reports highlights that funds received through donations (including individuals, CSR programs, and Indian foundations) for three financial years (FY 2020–2021, 2021–2022, and 2022–2023) were INR 409 M, INR 681 M, and INR 748 M respectively. Each year has witnessed a significant increase of fund inflow through donations indicating a strong relationship of faith built with donors, thus indicating sustainability. Another critical aspect of philanthropy that has driven sustainability is the operational efficiency, governance, and transparency of the institutions that influences donor trust, relationship, and retention.

#### Increased Government Support

**Fig. 4:**
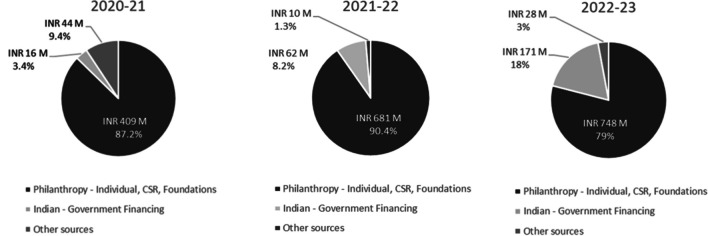
Sources of funds for Sai Sanjeevani Hospitals for 3 financial years. INR - Indian Rupee, M - Million

With regard to SSSSH, support from the governments increased from 3.4% of all fund sources on 2020–2021 to 18% in 2022–2023, thus diversifying the risks from the philanthropy mode of funding, strengthening the model (Fig. 4).

##### Social impact

Since 2012, SSSSH have made a significant social impact through their steadfast commitment to the tenet of ‘one world-one family’. This visionary approach has not only transformed lives but has also set a precedent for global healthcare, with compassion and inclusivity at its core.

At a macro level, the key impact areas of the hospitals are:

1. Establishment and running of three Centres for Child Heart Care for 11 years
2. Establishment and running of five Mother & Child hospitals for rural pregnant women for 3 years
3. Over 29,000 totally free of cost child heart surgeries and interventions in 11 years
4. Technical expertise and system strengthening support to address the burden of CHDs in LMICs by helping set up two philanthropy-based paediatric cardiac hospitals in the Fiji Islands and Sri Lanka.

While the impact achievements of Sai Sanjeevani Hospitals, through its free of cost model, are undeniable, it is crucial for us to look beyond the statistics and delve into the profound impact on social values and the lives of families benefiting from the provision of free healthcare.

An independent third-party study was conducted by the Social Audit Network [47], India, to measure the impact of Sai Sanjeevani Hospitals with a societal perspective for the period Apr 2018–Mar 2021, defined as all aspects of affordability, acceptability, and social return.

#### Affordability

**Fig. 5:**
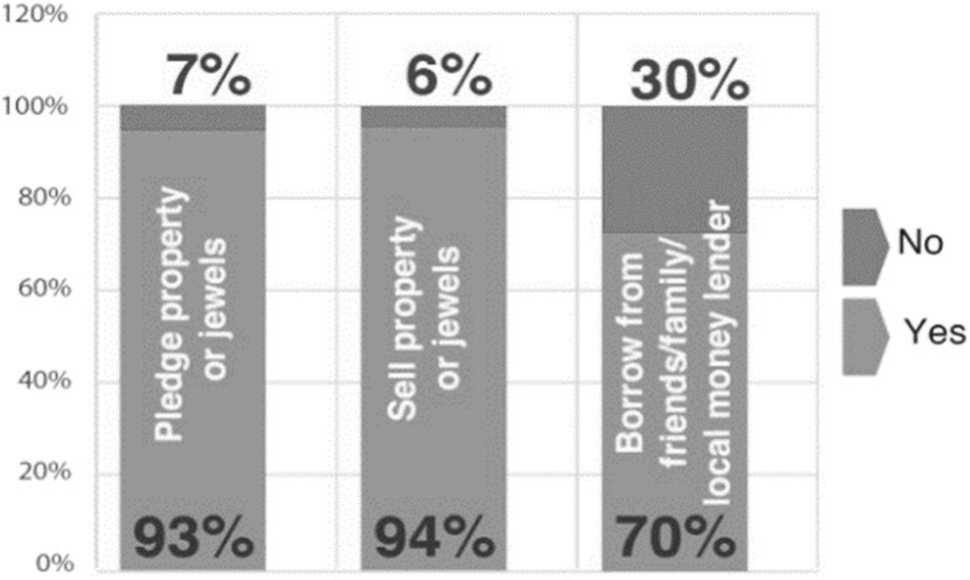
Feedback from beneficiary families on the potential financial risk averted

The study reported costs of paediatric cardiac surgeries across 12 states of India varying from INR 175,000 to INR 425,000 (USD 2000 to USD 5000). While at Sai Sanjeevani, none of the parents had to pay anything for their child’s treatment, without which they would have been driven to poverty by selling their property and other movable assets or taking loans from friends, family, or money lenders (Fig. 5).

#### Socioeconomic profile

Seventeen percent of the beneficiaries were reported to be from the most underprivileged socioeconomic strata of the society based on the Kuppuswamy Scale [48]. The majority of patients served report a monthly salary of between INR 7887 and INR 13,160 (USD 100–USD 150).

#### Impact on child health

**Fig. 6:**
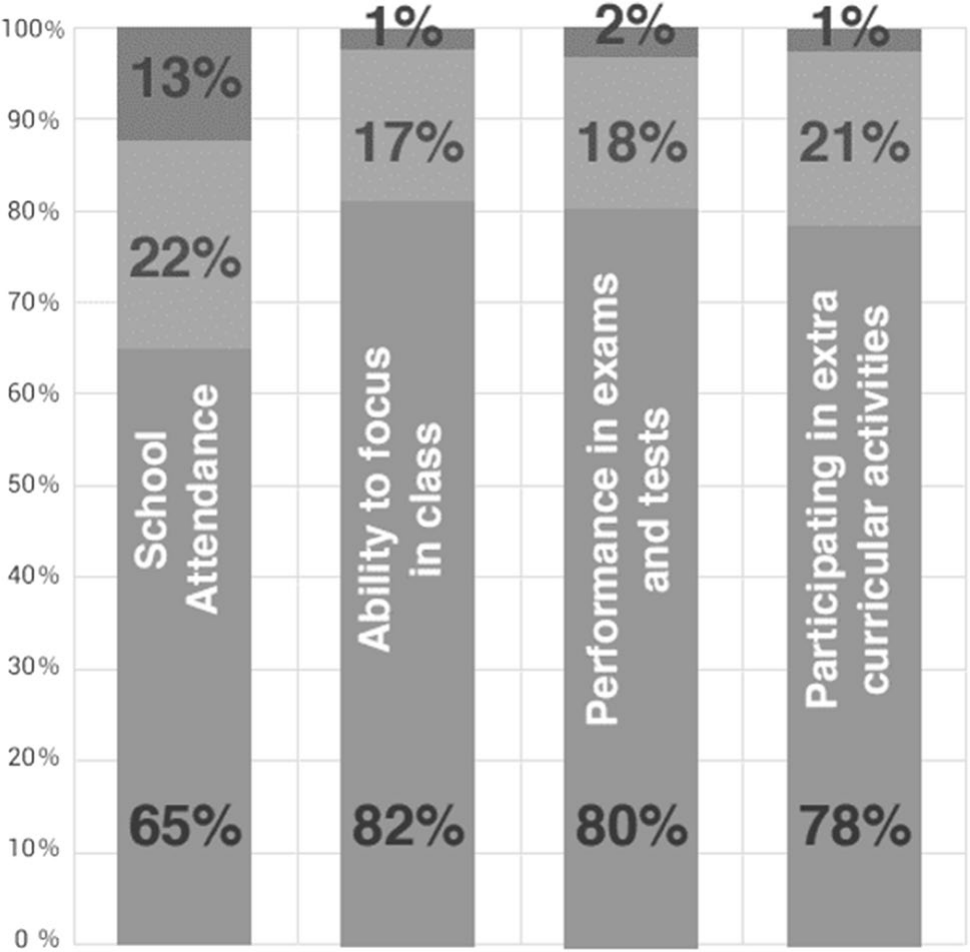
Feedback from beneficiary families on improvement in their child’s health post-surgery

Over 81% of families reported a remarkable level of improvement in the symptoms of their children. The majority of parents also reported a significant improvement in school attendance, performance, and participation (Fig. 6).

#### Impact on families

**Fig. 7:**
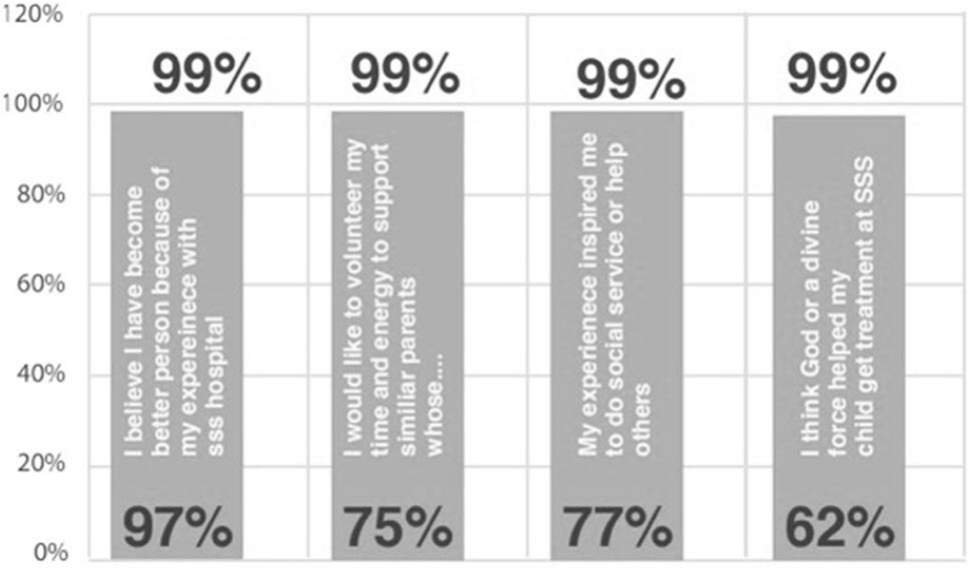
Feedback from beneficiary families on impact of Sanjeevani Hospitals on them

The hospitals have not only delivered ‘the gift of life’ to thousands of children—our future generation—but has also offered joy to thousands of parents and families since its beginnings, thus transforming into institutions spreading health and goodness in the society (Fig. 7).

#### Operational sustainability

Continued vision and nurturing young organisation leaders living the values of the institutions were highlighted as key success factors of the model [40]. The study also reported the medical, nursing, and paramedical training programs in paediatric cardiac care contributing back to the operational sustainability of the Sai Sanjeevani model, thus making highly specialised skills available for current and new centres for Child Heart Care.

#### Social return of investment (SROI)

SROI generates a benefit to cost ratio for organisations to communicate the value-add of their intervention and highlights how social change is created. Considering numerous parameters such as access to effective healthcare, financial benefit to families, savings for beneficiary families, and improved education opportunities as key benefits, the SROI of the SSSSH was reported to be 16.2. Thus, each operation conducted on a child at SSSSH generates a social value 16.2 times more than what was invested.

#### Global impact

Over the last decade, SSSSH have emerged as global models of Child Heart Care for replication by developing models, focusing on quality care and efficient operations, and serving the underserved. Apart from treating over 145 patients from 17 developing countries who have sought care at the Sai Sanjeevani Hospitals in India, the hospitals have conducted 12 international humanitarian missions for Child Heart Care in the Fiji Islands, Sri Lanka, Nigeria, and Malaysia, resulting in training local specialists, nursing, and paramedical teams.

In order to contribute to global capacities in terms of increase in centres for child heart care, Sai Sanjeevani Hospitals, India, over the last 5 years, have provided training and system strengthening support to organisations in the Fiji Islands and Sri Lanka to set up independent centres for Child Heart Care to provide free of cost screening and outpatient and surgical care to children with CHDs from the region.

- *Sri Lanka*—The SSSSH in Sri Lanka was established in Batticaloa in 2022 in association with the Karuna Nilayam Foundation to address adult and congenital heart conditions. Since commencement up to Jan 2024, the hospital has managed 21 CHD and 36,099 adult outpatients and conducted 11 paediatric cardiac surgeries and 537 adult cardiac interventions, all totally free of cost.
- *Fiji Islands*—The Sri Sathya Sai Sanjeevani Children’s hospital in the Fiji Islands was established in Suva in 2022 by Sai Prema Foundation Fiji to address CHD. Since commencement and until January 2024, the hospital has managed over 14,500 CHD outpatients and conducted 260 paediatric cardiac surgeries, all totally free of cost. The hospital provides free surgeries for children with CHD not only in Fiji, but all the surrounding South Pacific Islands as there is no other facility in the Pacific Islands.

## Conclusion

The global burden of CHDs demands urgent attention, particularly for low-middle income countries that face affordability and accessibility challenges. The assertion of this paper is that free care in Child Heart Care is no longer a choice. The case of SSSSH in India that run largely through philanthropy exemplifies collaborations between governments, institutions, and individuals bonded by societal values and provide a possible approach for international and social organisations in low-middle income countries seeking to commence and offer free CHD care through a sustained philosophy. Redefining sustainability as a composite result of both social impact and economics, the paper also marks a departure from a purely financial perspective of sustainability.

The success of the Sri Sathya Sai Sanjeevani model underscores the relevance of adopting a holistic approach, including state-of-the-art infrastructure, service mapping, advocacy, screening, training and education, research and phased growth to ensure sustained care provision, scalable philanthropy, and continued collaborations [49, 50]. The model also highlights the need for organisations to focus on operational efficiencies, transparency, and governance for donor retention, a key contributor to sustainable philanthropy. Collaborations have in them the intrinsic power of partnerships which would be the main driving and success factor towards developing a global strategy for philanthropy driven CHD care in LMICs, as is seen in the cases of Sri Lanka and Fiji.

The paper suggests that providing free CHD surgeries through sustained philanthropy is a possibility and recognises the need for more research to evaluate the GIVE model, relevance of the discussed sustainability model in different geographies and implementation models, success factors for sustained philanthropy, relevance of philanthropy in low-income countries, and global examples on collaborations with regard to CHD care. In the pursuit of this noble goal, it is time for the world to unite, rise to the challenges, and pave the way for a future where every child, regardless of socioeconomic background, can receive the life-saving care they deserve.

## Data Availability

Not applicable as it is a review article. However, the authors confirm that the data supporting the findings of this study are available within the article or its supplementary material.
